# Obesity and the Odds of Weight Gain following Androgen Deprivation Therapy for Prostate Cancer

**DOI:** 10.1155/2014/230812

**Published:** 2014-04-22

**Authors:** Lior Z. Braunstein, Ming-Hui Chen, Marian Loffredo, Philip W. Kantoff, Anthony V. D'Amico

**Affiliations:** ^1^Harvard Radiation Oncology Program, Brigham and Women's Hospital, ASB-I Radiation Oncology L2, 75 Francis Street, Boston, MA 02115, USA; ^2^Department of Statistics, University of Connecticut, 215 Glenbrook Road, U-4120, Storrs, CT 06269-4120, USA; ^3^Lank Center for Genitourinary Oncology, Dana-Farber Cancer Institute, Mailstop Dana 1230, 450 Brookline Avenue, Boston, MA 02215, USA; ^4^Department of Radiation Oncology, Dana-Farber/Brigham and Women's Hospital, ASB-I Radiation Oncology L2, 75 Francis Street, Boston, MA 02115, USA

## Abstract

*Background*. Increasing body mass index (BMI) is associated with increased risk of mortality; however, quantifying weight gain in men undergoing androgen deprivation therapy (ADT) for prostate cancer (PC) remains unexplored. * Methods*. Between 1995 and 2001, 206 men were enrolled in a randomized trial evaluating the survival difference of adding 6 months of ADT to radiation therapy (RT). BMI measurements were available in 171 men comprising the study cohort. The primary endpoint was weight gain of ≥10 lbs by 6-month followup. Logistic regression analysis was performed to assess whether baseline BMI or treatment received was associated with this endpoint adjusting for known prognostic factors. * Results*. By the 6-month followup, 12 men gained ≥10 lbs, of which 10 (83%) received RT + ADT and, of these, 7 (70%) were obese at randomization. Men treated with RT as compared to RT + ADT were less likely to gain ≥10 lbs (adjusted odds ratio (AOR): 0.18 [95% CI: 0.04–0.89]; *P* = 0.04), whereas this risk increased with increasing BMI (AOR: 1.15 [95% CI: 1.01–1.31]; *P* = 0.04). * Conclusions*. Consideration should be given to avoid ADT in obese men with low- or favorable-intermediate risk PC where improved cancer control has not been observed, but shortened life expectancy from weight gain is expected.

## 1. Introduction

The addition of androgen deprivation therapy (ADT) to external beam radiation therapy (RT) has been shown to prolong overall survival in men with intermediate or high-risk prostate cancer (PC) enrolled on several randomized controlled trials [1–7]. Despite this well-established survival benefit, it is known that ADT has significant side effects that adversely affect quality of life [3, 5, 8]. These side effects include hot flashes, gynecomastia, decreased libido, nipple sensitivity, and decreased metabolism [9–14]. Moreover, ADT use in excess of 1 year has also been shown to increase the risk of osteoporosis [15, 16], diabetes [17–20], and cardiovascular disease [18, 21, 22].

With regard to decreased metabolism, several investigators have shown that ADT causes a decrease in lean body mass with a concomitant increase in total body fat of up to ~10% [14, 16]. Of note, the increase in fat mass appears to be distributed centrally about the abdominal compartment [23]. Based on these findings, men are commonly advised that weight gain is possible during ADT; however, the degree to which weight gain occurs and risk factors associated with weight gain are not well documented. Given the known association of an elevated BMI for men who are overweight (BMI 25 to 29.9 kg/m^2^) or obese (BMI ≥ 30 kg/m^2^) and an increased risk of mortality [24], understanding weight changes during ADT use is important when counseling men about diet and exercise during ADT in order to minimize weight gain and thereby avoid increasing the risk of mortality.

Therefore, the purpose of this study was to use data from a prospective randomized trial in order to ascertain clinical factors at randomization associated with significant weight gain (≥10 pounds) following the completion of RT and ADT [3]. In addition, we quantified weight gain across randomized treatment arms and within BMI categories as measured at baseline in order to quantify the effect of ADT on weight gain within each BMI category using the radiation-only arm as a control.

## 2. Methods

### 2.1. Patient Population and Treatment

Between December 1, 1995, and April 15, 2001, 206 men were enrolled in a prospective randomized trial evaluating the impact on the survival of adding 6 months of combined ADT to ~70 Gy RT [3]. Prior to randomization, patient age, prostate biopsy results, Gleason score, serum prostate-specific antigen level (PSA), digital rectal exam findings (DRE), and adult comorbidity evaluation 27 (ACE-27) scores were ascertained and recorded. Of 206 men, 11 did not have a body mass index (BMI) measured at randomization and 24 did not have BMI measured at the endpoint, leaving 171 patients who formed the current study cohort. Patients were randomized to RT alone consisting of 3-dimensional conformal RT to ~70 Gy or to the same RT regimen with 2 months of neoadjuvant, concurrent, and adjuvant combined ADT totaling 6 months and composed of a luteinizing hormone-releasing hormone (LHRH) agonist and the antiandrogen flutamide. This secondary analysis of the primary study was approved by the institutional review board of the Dana-Farber/Harvard Cancer Center; informed consent was obtained for the primary study. There is no funding for this secondary analysis.

### 2.2. Assessment of Weight Gain at End of Treatment

Following the completion of RT with or without 6 months of combined ADT, men were seen at approximately 6 months after randomization. At each followup, a digital rectal examination, serum PSA, and weight measurement were obtained. The scale used to measure patients at this 6-month followup was the same as at randomization and the difference in weight in pounds was ascertained and recorded between the two time-points.

### 2.3. Statistical Methods 

#### 2.3.1. Distribution and Comparison of Clinical Factors of the Study Cohort Stratified by Randomized Treatment Arm

Clinical characteristics at baseline were enumerated and compared across randomized treatment arms. For the continuous covariates of BMI, PSA, and age, the nonparametric Wilcoxon test [25, 26] was used to compare the distributions of these factors across randomized treatment arms. A Mantel-Haenszel chi-square metric [27, 28] was used to compare the distribution of categorical covariates including highest biopsy Gleason score, 2009 AJCC tumor (T) category [29], and ACE-27 comorbidity score across randomized treatment arms.

#### 2.3.2. Logistic Regression Analysis

The primary endpoint of this study was whether the patient gained ≥10 lbs by the 6-month follow-up point after randomization. Univariable and multivariable logistic regression [30] analysis was performed to assess whether baseline BMI or treatment received was associated with this endpoint adjusting for comorbidity and known PC prognostic factors. Time zero was the date of randomization. BMI, PSA, and age were treated as continuous covariates, whereas treatment arm, Gleason score, tumor category, and ACE-27 score were considered as categorical covariates in the model. The baseline group for the categorical variables included the RT with ADT treatment arm, Gleason score ≤ 6, tumor category 1 (T1), and ACE-27 with no or minimal comorbidity, respectively. Adjusted odds ratios and their associated 95% confidence intervals and *P* values were calculated. Two-sided *P* values ≤ 0.05 were considered statistically significant. SAS version 9.3 was used for all statistical analyses.

#### 2.3.3. Distribution of Weight Gain 6 Months following Randomization, Stratified by Treatment Received and BMI

The distribution of the 85 and 86 men, who underwent RT or RT and ADT, respectively, and experienced ≥10 lbs weight gain versus <10 lbs, 6 months following randomization, stratified by well-defined BMI cut-points for normal weight, overweight, and obese, was compared using Fisher's exact test [31].

## 3. Results 

### 3.1. Distribution and Comparison of Clinical Factors of the Study Cohort Stratified by Randomized Treatment Arm

Table 1 illustrates the distribution of clinical factors stratified by randomized treatment arm (RT versus RT + ADT). As expected, given the randomization, all factors including BMI, PSA, patient age, Gleason score distribution, and ACE-27 comorbidity score were not significantly different between the two treatment arms (*P* value for each factor ≥ 0.11). Of note, the median BMI and its distribution were nearly equivalent between the two arms at baseline (27.44 kg/m^2^ [IQR = 25.58, 30.23] versus 27.35 kg/m^2^ [IQR = 24.68, 30.99]; *P* = 0.73).

### 3.2. Logistic Regression Analysis

By the 6-month followup, 12 men were observed to have gained ≥10 lbs of which 10 (83%) were treated with RT and ADT, and 7 (70%) were obese at the time of randomization. For these 7 men, the median increase in BMI was 5.21% (range: 3.60%–6.37%). As shown in Table 2, men treated with RT as compared to RT and ADT were significantly less likely to experience a weight gain of ≥10 lbs (AOR: 0.18 [95% CI 0.04–0.89]; *P* = 0.04), whereas this risk was increased with increasing BMI (AOR: 1.15 [95% CI 1.01–1.31]; *P* = 0.04). No other clinical factors were found to be significantly associated with this endpoint.

### 3.3. Distribution of Men Observed to Experience at Least a 10 Pound Weight Gain 6 Months following Randomization, Stratified by Treatment Received and BMI Category

Table 3 illustrates the significant findings of the logistic regression multivariable analysis. Specifically, men treated with RT and ADT and who were obese at randomization were significantly more likely to gain ≥10 lbs as compared to <10 lbs by 6 months following randomization. These respective percentages were 70% versus 22%; *P* = 0.006. However, this significant trend was not noted for men who were obese at randomization and underwent RT alone where the respective values were 0% and 28%; *P* = 0.45. Of patients with a normal BMI at the time of enrollment, only 1 out of 18 men (6%) who received RT alone and 2 out of 24 men (8%) who received RT and ADT gained ≥10 lbs (Fisher's exact test, *P* = 1.00), suggesting no significant risk to normal-weight men of becoming overweight from treatment with ADT.

## 4. Discussion

In this study, we observed that men at highest risk of ≥10 lbs weight gain following RT and 6 months of ADT were those who were obese at the outset of treatment. Specifically, for every 1 unit increase in BMI, there was a 15% increase in the odds of gaining at least 10 pounds by the 6-month follow-up visit and the median increase in BMI was 5.21%. Given the established association of increased mortality with additional weight gain in obese men [24], these findings suggest that some obese men may be at risk for a shortened survival with ADT use. Therefore, the clinical significance of this finding suggests taking a measured risk/benefit approach when deciding on ADT use in obese men. In particular, this consideration becomes extremely pertinent for obese men in whom ADT use may have little or no impact on reducing the risk of prostate cancer-specific mortality (PCSM) but can shorten life expectancy.

Several points require further clarification. First, weight gain in obese patients is known to reduce both quality of life (QoL) and longevity [24, 32, 33], in part due to increased risk of cardiovascular events [34–36] and the sequelae of diabetes [37, 38]. Yet, there is no proven benefit for the use of ADT in men with low risk PC despite its frequent use (up to 19%) documented within a large observational database between 1989 and 2002 (UCSF Cancer of the Prostate Strategic Urologic Research Endeavor—CaPSURE) [39].

Specifically, ADT has been used in men with benign prostatic hyperplasia and low risk PC who are not candidates for brachytherapy due to pubic arch interference as determined at the time of volume study [40, 41]. In such men, ADT has no proven benefit on reducing PCSM [5], but the results of the current study show that ADT use can lead to significant weight gain in men who are already obese, which places them at higher risk of earlier morbidity and mortality because of further increases in their BMI [13, 24, 33]. Second, recent literature suggests that men with favorable-intermediate risk prostate cancer [42] may not have a reduction in the risk of PCSM from the addition of ADT to high dose RT [43]. Moreover, randomized controlled trials that have established a survival benefit when ADT is added to RT in men with unfavorable-intermediate or high-risk prostate cancer did not have a prerandomization stratification by comorbidity, and a postrandomization analysis by comorbidity at randomization found no survival benefit for the addition of ADT to RT [44]. Therefore, by applying similar reasoning, obese men with favorable-intermediate risk PC may not benefit from ADT use and may also be at risk for shortened survival and declining quality of life, without reduction in PSA recurrence, metastasis, or death from PC from adding ADT to RT.

Therefore, ADT use should be discouraged in obese men with low- or favorable-intermediate risk PC. Moreover, future studies should employ a validated QoL metric [45] and a measurement of PC-specific and overall survival in obese men with unfavorable-intermediate and high-risk PC to ascertain the risk/benefit ratio of adding ADT to RT in obese men.

A limitation of the current analysis is the relatively small event rate (*N* = 12) of a ≥10 lbs weight gain following RT and 6 months of ADT. Therefore, validation of these results by other investigators is needed. However, while the overall event rate was small, the proportion of obese men who achieved this endpoint was substantial at 70%. Moreover, given that the proportion of men in the United States who are obese and over the age of 50 is increasing [32], and with the use of PSA screening, the proportion of men with low- or favorable-intermediate risk PC has also been increasing [46–48], and the potential negative impact of ADT use on life expectancy would be expected to also increase if ADT use is continued in these men. A strength of this study is that the data are from a prospective randomized trial. As a result, the radiation control arm is available for calculating the increased odds of weight gain from the multivariable logistic regression analysis for men who were randomized to receive RT and 6 months of ADT. Therefore, other reasons for weight gain besides ADT use are controlled for by the study design, lending additional support to both ADT and increasing BMI being the drivers of the weight gains observed.

In conclusion, obese men are at increased risk for ≥10 lbs weight gain by the end of 6 months of ADT, prompting serious consideration to limiting or avoiding the use of ADT in these men with low- or favorable-intermediate risk PC where improvement in cancer control has not been observed but a shortened life expectancy from further weight gain may be expected.

## Figures and Tables

**Table 1:** Distribution and comparison of clinical factors at randomization of the study cohort stratified by randomized treatment arm.

Clinical factor	Treatment with RT *N* = 85	Treatment with RT and AST *N* = 86	*P* value
Median BMI (IQR)	27.44 kg/m^2^ (25.58, 30.23)	27.35 kg/m^2^ (24.68, 30.99)	0.73
Median PSA (IQR)	11.54 ng/mL (7.70, 16.40)	10.85 ng/mL (7.50, 15.51)	0.38
Median Age (IQR)	73.36 (70.82, 76.15)	72.12 (69.07, 74.71)	0.11
T1	37 (44%)	46 (53%)	0.19
T2	48 (56%)	40 (47%)	
Gleason score 6 or less	26 (31%)	25 (29%)	0.92
7	45 (53%)	49 (57%)	
8 to 10	14 (16%)	12 (14%)	
No or minimal cm	65 (76%)	65 (76%)	0.89
Moderate to severe cm	20 (24%)	21 (24%)	

RT indicates radiotherapy; ADT: androgen deprivation therapy; BMI: body mass index; IQR: interquartile range; PSA: prostate-specific antigen; cm: comorbidity.

**Table 2:** Univariate and multivariate odds ratios for the risk of ≥10 lbs weight gain 6 months after randomization for each clinical factor.

Clinical factor	Number of men	Number of men who gained ≥10 lbs by EOT	Univariable analysis		Multivariable analysis	
			OR (95% CI)	*P* value	AOR (95% CI)	*P* value
RT	85	2	0.18 (0.04, 0.86)	0.03	0.18 (0.04, 0.89)	0.04
RT + AST	86	10	1 (Ref)	—	1 (Ref)	—
BMI increase per kg/m^2^	171	12	1.18 (1.05, 1.33)	0.01	1.15 (1.01, 1.31)	0.04
PSA increase per ng/mL	171	12	0.98 (0.90, 1.06)	0.58	0.97 (0.87, 1.08)	0.57
Age	171	12	0.93 (0.85, 1.02)	0.14	0.95 (0.85, 1.06)	0.38
Gleason score 8 to 10	26	3	2.09 (0.39, 11.15)	0.39	1.17 (0.17, 7.93)	0.87
7	94	6	1.091 (0.26, 4.56)	0.90	0.55 (0.10, 3.06)	0.49
6 or less	51	3	1 (Ref)	—	1 (Ref)	—
T2	88	8	1.98 (0.57, 6.82)	0.28	1.99 (0.49, 7.99)	0.33
T1	83	4	1 (Ref)	—	1 (ref)	—
Mod to Sev cm	41	5	2.44 (0.73, 8.15)	0.15	2.11 (0.54, 8.26)	0.28
No or min cm	130	7	1 (Ref)	—	1 (Ref)	—

RT indicates radiotherapy; ADT: androgen deprivation therapy; BMI: body mass index; PSA: prostate-specific antigen; cm: comorbidity; OR: odds ratio; CI: confidence interval; AOR: adjusted odds ratio; EOT: end of treatment.

**Table 3:** Distribution of weight gain 6 months following randomization, stratified by treatment received and body mass index.

	RT only (*N* = 85)		RT + ADT (*N* = 86)	
	Weight change 6 months following randomization			
	<10 lbs (*N* = 83)	≥10 lbs (*N* = 2)	<10 lbs (*N* = 76)	≥10 lbs (*N* = 10)
BMI (range in kg/m^2^)	(Range −13 to +9; median −1 lb)	(Range 10 to 13; median +11.5 lbs)	(Range −11 to +8; median +1 lb)	(Range 10 to 17 lbs; median +11.25 lbs)
Normal (18.5–24.9)	17 (20%)	1 (50%)	22 (29%)	2 (20%)
Overweight (25.0–29.9)	43 (52%)	1 (50%)	37 (49%)	1 (10%)
Obese (≥30.0)	23 (28%)	0 (0%)	17 (22%)	7 (70%)
	Fisher's exact test *P* value = 0.45		Fisher's exact test *P* value = 0.006	

RT indicates radiotherapy; ADT: androgen deprivation therapy; BMI: body mass index.
